# Variability of the response of human vaginal *Lactobacillus crispatus* to 17β-estradiol

**DOI:** 10.1038/s41598-021-91017-5

**Published:** 2021-06-01

**Authors:** Maximilien Clabaut, Amine M. Boukerb, Amine Ben Mlouka, Amandine Suet, Ali Tahrioui, Julien Verdon, Magalie Barreau, Olivier Maillot, Agathe Le Tirant, Madina Karsybayeva, Coralie Kremser, Gérard Redziniak, Cécile Duclairoir-Poc, Chantal Pichon, Julie Hardouin, Pascal Cosette, Sylvie Chevalier, Marc G. J. Feuilloley

**Affiliations:** 1 Laboratory of Microbiology Signals and Microenvironment (LMSM EA 4312), Rouen Normandie Université, 55 rue Saint-Germain, 27000 Evreux, France; 2 Laboratory «Polymères, Biopolymères, Surfaces» (UMR 6270 CNRS), Proteomic Platform PISSARO University of Rouen Normandy, Mont-Saint-Aignan, France; 3 Centre de Biophysique Moléculaire, UPR4301 French National Centre for Scientific Research, Orléans, France; 4 Laboratoire EBI, UMR CNRS 7267, Université de Poitiers, Poitiers, France; 5 Seqens Cosmetics, Porcheville, France; 6 Remedials Laboratory, Paris, France; 7 GymoPharm, Longjumeau, France; 8 Cosmetic Inventions, Antony, France

**Keywords:** Microbiology, Physiology

## Abstract

We previously showed that the physiological concentration of 17β-estradiol in the vaginal environment is sufficient to affect the membrane dynamics and adhesion phenotype of the *Lactobacillus crispatus* strain CIP104459. However, *L. crispatu*s is a heterogeneous species. Here, we investigated the effect of 17β-estradiol on the recently isolated *L. crispatus* vaginal strain V4, related to a cluster distant from CIP104459 and at the limit of being a different subspecies. Grown in the same medium, the two strains expressed a highly similar pool of proteins. However, in contrast to CIP104459, *L. crispatus* V4 showed high aggregation potential and 17β-estradiol promoted this phenotype. This effect was associated with large changes in cell-surface polarity and Lewis acid/base properties. In addition, we observed no effect on the membrane dynamics, contrary to CIP104459. These results can be explained by differences in the properties and organization of the S layer between the two strains. However, as for CIP104459, 17β-estradiol increased biosurfactant production of *L. crispatus* V4 and their adhesion to vaginal cells. This suggests that 17β-estradiol agonists would be valuable tools to favor a stable re-implantation of *L. crispatus* in the vaginal mucosa.

## Introduction

The vaginal microbiota of healthy non-pregnant reproductive aged women is characterized by a limited number of bacterial species, most of which belong to the *Lactobacillus* genus, including *L. crispatus*, *L. gasseri*, *L. iners* and *L. jensesenii*^1,2^. Among them, *L. crispatus* is the most abundant^3^. However, the *L. crispatus* species is itself heterogeneous, as shown by whole genome sequencing^4^. In addition, there appears to be competition between *L. crispatus* variants, resulting in a mutual exclusion process and consequently to highly homogeneous and almost clonal populations of the same strain in the vaginal niche^4^. Lactobacilli, and particularly *L. crispatus*, are known to be beneficial microorganisms that can antagonize a large range of pathogens. However, as recently demonstrated, such antimicrobial activity is also strain dependent^5^, possibly explaining the variability of the protection provided by *L. crispatus* against bacterial vaginosis^6^.

These observations are essential, as various strategies have been developed to maintain or re-implant *L. crispatus* in the vaginal microbiota, particularly in post-menopausal women, but are often not successful over a long time^7^. A possible reason is the direct effect of estradiol on *L. crispatus*. As recently demonstrated using the reference strain *L. crispatus* CIP104459 from the Pasteur Institute Collection, sub-micromolar concentrations of 17β-estradiol affect bacterial membrane homeostasis and promote biosurfactant production, leading to increased adhesion to vaginal epithelium mucosa cells^8^. Data has suggested that these effects are mediated by signal transduction and a putative 17β-estradiol sensor protein, the membrane lipid rafts-associated SPFH domain-containing protein, showing homology with the eukaryotic estrogen-related receptor gamma (ERR3) and the estradiol binding protein prohibitin-2 (PHB2), was identified in the *L. crispatus* CIP104459 genome^8^. However, our knowledge of the direct impact of estradiol on *Lactobacillus* physiology is still very limited, and particularly the potential differences in the responses of *L. crispatus* variants. Addressing this question may be of high importance, as vaginal *L. crispatus* variants express different glycosyltransferase genes, which could strongly affect their affinity to glycogen and the vaginal mucosa^9^. In addition, the response to host factors can differ between strains and even ribotypes, as demonstrated for skin associated bacteria^10^.

Here, we took advantage of the identification and availability of the draft genome sequence of a new vaginal strain of *L. crispatus*, designated as V4^11^, to compare its genome to that of other *L. crispatus* strains, particularly that of the *L. crispatus* CIP104459 Pasteur reference strain^12^. The response of *L. crispatus* V4 to 17β-estradiol was subsequently studied using proteomic, physiological, physicochemical, morphological, and cell binding approaches.

## Results

### Phylogenetic relationships of *L. crispatus* V4 with other vaginal *Lactobacillus*

**Figure 1:**
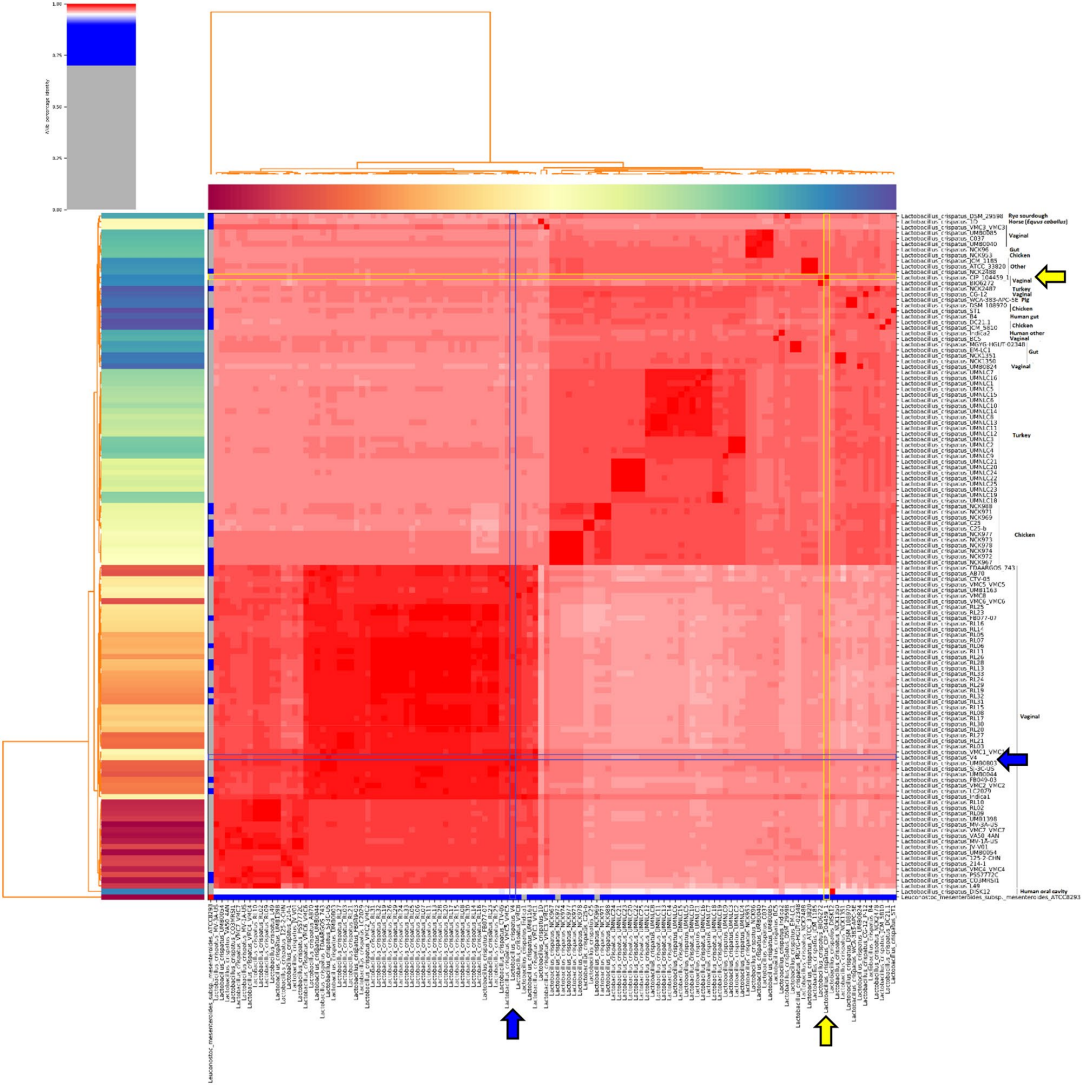
Heatmap and dendrogram of average nucleotide identity (ANI) illustrating the phylogenetic relationships based on the percentage identity [high (red) to low (blue/grey)] shared between the whole sequence of *Lactobacillus crispatus* V4 (blue boxes and arrows) and 122 other *L. crispatus* isolates for which the genome sequences were accessible in the NCBI RefSeq database (accessed on 3rd November, 2020), including that of *Lactobacillus crispatus* CIP104459 from Clabaut et al*.*^12^ (yellow boxes and arrows). The phylogenic tree was rooted using the genome sequence of an unrelated bacterial species, presently *Leuconostoc mesenteroides* subspecies mesenteroides, ATCC8293.

**Figure 2:**
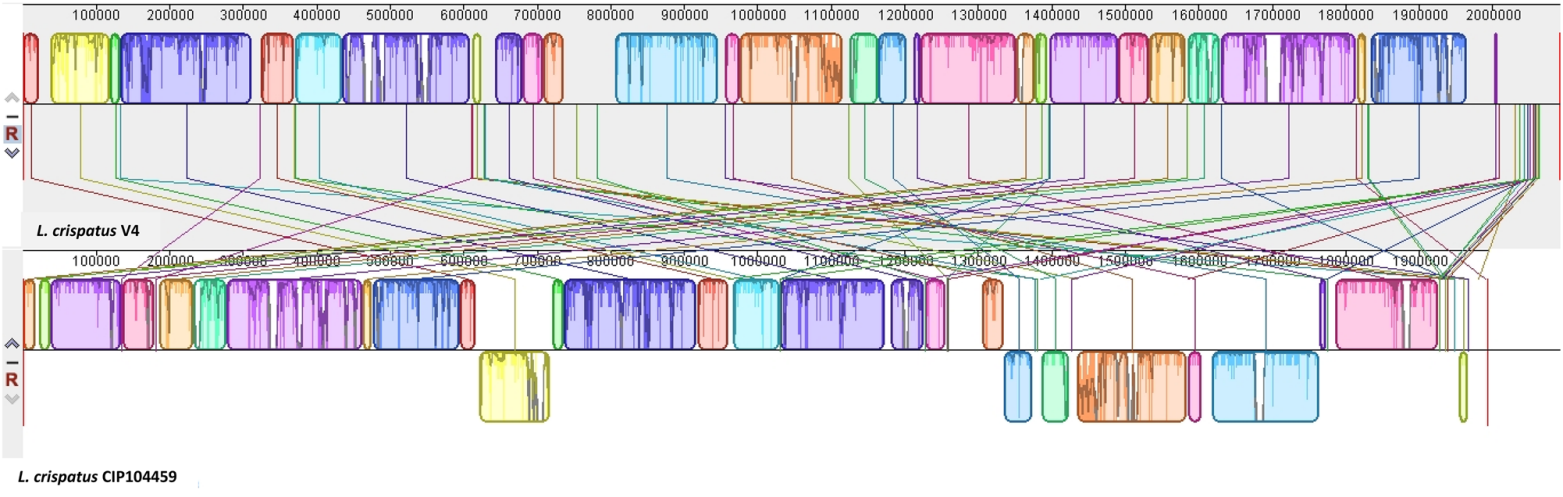
Comparative genome synteny between *Lactobacillus crispatus* V4 and *Lactobacillus crispatus* CIP104459. Homologous regions are identified as locally collinear blocks (LCBs). White regions correspond to sequences that are not aligned and probably contain sequence elements specific to a particular genome. Flipped blocks indicate a reverse complement (inverse) orientation.

We compared the sequenced genome of *L. crispatus* V4^11^ to that of 122 *L. crispatus* strains for which the genomes were accessible in the RefSeq NCBI Reference Sequence Database (accessed on November 3rd, 2020). The percentage of shared average identity of the *L. crispatus* V4 core genome with that of *L. crispatus* CIP104459, for which the response to 17β-estradiol was previously studied^8^, was only 97.42% (Suppl. Table 1). A genome-to-genome distance analysis indicated an in silico DNA-DNA hybridization value (isDDH) of 78.70%. Both results suggest a significant genomic distance within the same bacterial species. Indeed, the two strains are related to different phylogenetic clusters (Fig. 1). *L. crispatus* CIP104459 is close to human and animal strains from diverse origins, whereas *L. crispatus* V4 appears to be related to typical human vaginal strains. We aligned the whole genomes of *L. crispatus* V4 and *L. crispatus* CIP104459, which contain 2178 and 2034 open reading frames (ORFs), respectively (Fig. 2). The core genome shared by the two strains is limited to 1638 ORFs (corresponding to 77.96% of the average complete genomes), whereas a high number of genes are unique to *L. crispatus* V4 or CIP104459 (540 and 386, respectively). Noticeably, full-length copies of gene sequences similar to the *glgX* gene, encoding a glycogen debranching enzyme designated as a type 1 pullulanase, were present in both the genome sequences of *L. crispatus* V4 and CIP104459. After Prokka-based genome annotation and manual curation, the genome sequences of both strains also appeared to contain genes encoding other putative glycosyltransferases, including *csbB*, *epsH,* and *glyD,* in addition to *epsF* in *L. crispatus* V4 and *epsE1* in *L. crispatus* CIP104459.

### Comparison of the *L. crispatus* V4 and CIP104459 proteomes

We performed a proteomic analysis by nano LC–MS/MS on total proteins produced by the bacteria. Under our experimental conditions, 420 ± 6 different proteins were identified from *L. crispatus* V4 extracts. Proteins identified in the *L. crispatus* V4 core proteome (374) were related to 14 different functions, including DNA synthesis, transcription and regulation (25), translation, ribosomal structure and biogenesis (73), posttranslational modification, protein turnover, and chaperones (32), energy production and conversion (21), carbohydrate transport and metabolism (56), amino-acid transport and metabolism (46), nucleotide transport and metabolism (15), inorganic ion transport and metabolism (3), fatty-acid and phospholipid metabolism (11), secretion and export (1), membrane function (1), S layer, cell wall, and envelope biogenesis (16), and adaptation and protection (32). We also detected hypothetical proteins (42) (Suppl. Table 2). The total number of proteins extracted from *L. crispatus* CIP104459 was quite similar (409 ± 4). However, only 246 (out of 420, i.e., 58.5%) proteins identified in *L. crispatus* V4 were found in the *L. crispatus* CIP104459 proteome, whereas 332 proteins of CIP104459 (81.1%) were detected in the V4 proteome. Expression of the proteins identified in V4 and CIP varied widely between the two strains. Among the 332 proteins of the V4 proteome common to V4 and CIP104459, 226 showed a > twofold difference. Most (80.5%) were overexpressed in V4 (Suppl. Table 3^3^). Remarkably, only one protein involved in protein secretion, the dipeptide-binding protein DppE, was differentially expressed in V4 (× 6.41). Two membrane proteins were also overexpressed, particularly the oligopeptide-binding protein OppA (× 242.12). Envelope-associated proteins, such as FtsZ, also appeared to be upregulated in V4.

Quantification of proteins expressed by *L crispatus* V4 in the absence or presence of 17β-estradiol showed very limited differences. There were no differences in protein expression > twofold, considered to be the limit of significance, between *L. crispatus* V4 exposed or not to 10^–8^ M of 17β-estradiol and only one protein, elongation factor G (MHJPMHAL_00289), showed an increase > twofold after exposure to 10^–10^ M of 17β-estradiol (+ 2.079).

### 17β-estradiol affects the 1*L. crispatus* V4 aggregation phenotype

**Figure 3:**
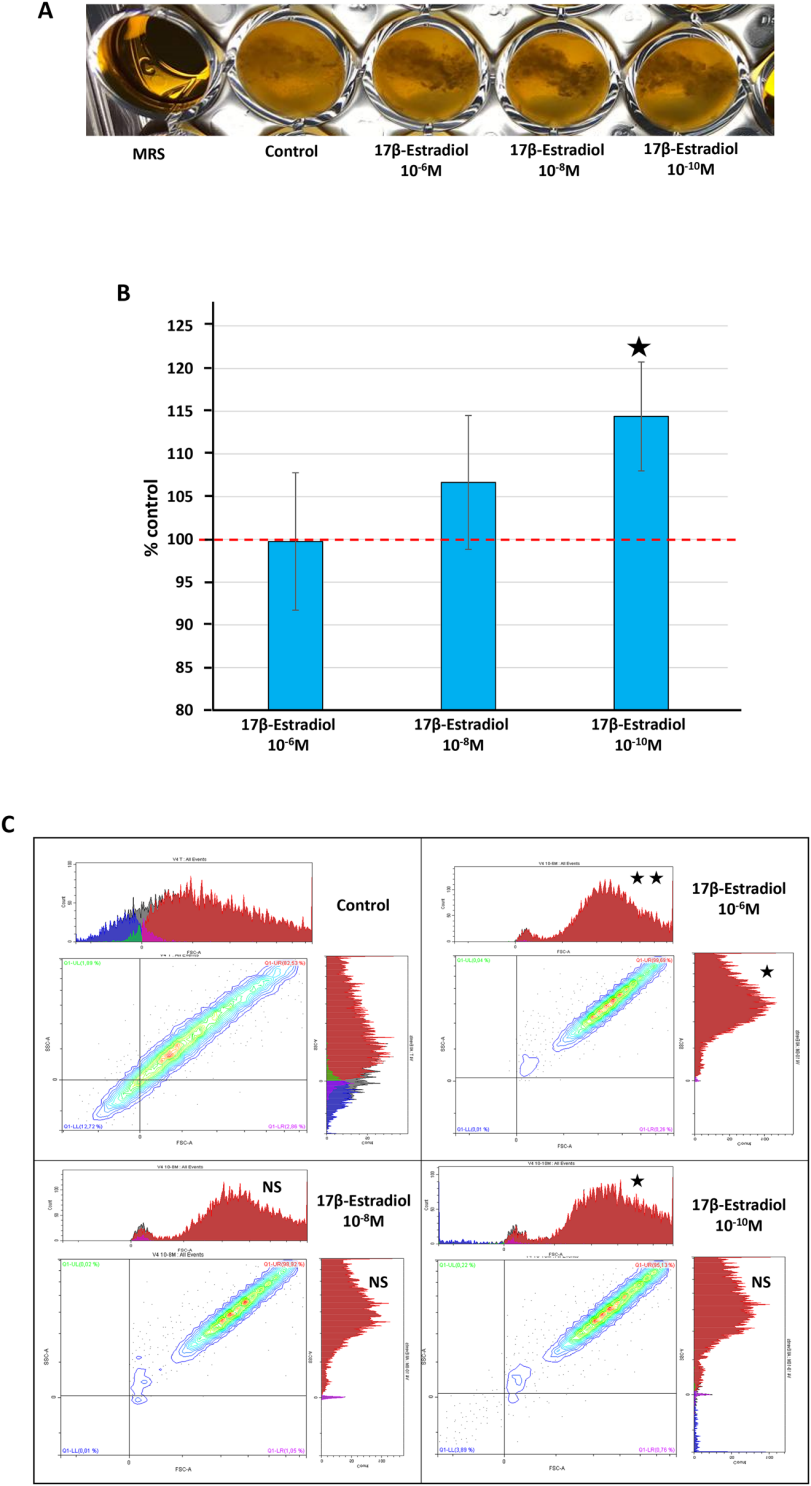
Effect of 17β-estradiol on the aggregation potential of *Lactobacillus crispatus* V4. (**A**) Typical aspect of *L. crispatus* V4 cultures, showing the formation of dense aggregates in the presence of 17β-estradiol (10^–6^, 10^–8^, and 10^–10^ M). (**B**) Quantification of aggregate formation by *L. crispatus* V4 grown in the presence of 17β-estradiol (10^–6^, 10^–8^, and 10^–10^ M), by the sedimentation technique. The red dotted line represents aggregate formation in control MRS medium. (**C**) Flow cytometry diagrams illustrating the effect of 17β-estradiol (10^–6^, 10^–8^, and 10^–10^ M) on the mean size and aggregation [X axis = forward scattering fraction (FSC)] and surface heterogeneity (Y axis = side scatter (SSC)) of the bacteria. Values are presented as the means ± SEM of a minimum of three independent studies (*NS* not significant, ^★^*p* < 0.05, ^★★^*p* < 0.01).

Preliminary studies showed that 10^–6^ to 10^–10^ M of 17β-estradiol had no effect on the growth kinetics of *L. crispatus* V4 (Suppl. Figure 1A). *Lactobacillus* can spontaneously form aggregates and *L. crispatus* is among the species with the highest potential^13^. The aggregation potential of *L. crispatus* V4 visibly increased after exposure to 17β-estradiol (Fig. 3A). We quantified the aggregation phenotype using the sedimentation technique^14^ (Fig. 3B), which showed an inverted dose-related effect for 17β-estradiol was observed. 17β-estradiol at 10^–6^ M had no detectable effect, whereas we observed a non-significant increase of the aggregation phenotype with bacteria exposed to 10^–8^ M 17β-estradiol. 17β-estradiol at 10^–10^ M led to significant stimulation of *L. crispatus* V4 aggregation, reaching + 114.4 ± 6% of the control (*p* < 0.05). We further investigated the aggregation phenotype of *L. crispatus* V4 by flow cytometry analysis (Fig. 3C). The mean size of detected structures was plotted on the horizontal axis (forward scattering fraction of the light, FSC) and the surface heterogeneity (granularity) on the vertical axis (side scatter light, SSC)^15^. Exposure of the bacteria to 10^–6^ M 17β-estradiol resulted in a significant increase in mean particles size (+ 20.76 ± 0.5% of the control; *p* < 0.01). The signal was divided between two peaks, the smaller one likely corresponding to isolated bacteria. In parallel, the surface heterogeneity of detected particles was higher than that of the control (*p* < 0.05). We observed the same tendency for bacteria exposed to 10^–8^ M 17β-estradiol and the FSC signal was divided between two peaks, as before, whereas the surface heterogeneity reverted to a unique group, but the differences were not statistically significant. Bacteria treated with 10^–10^ M 17β-estradiol at showed the same phenotype. The increase in the abundance of large particles (aggregates) was significant (*p* < 0.05), as observed with 10^–6^ M 17β-estradiol.

### 17β-estradiol affects the surface polarity and Lewis acid/base behavior of *L. crispatus* V4

**Figure 4:**
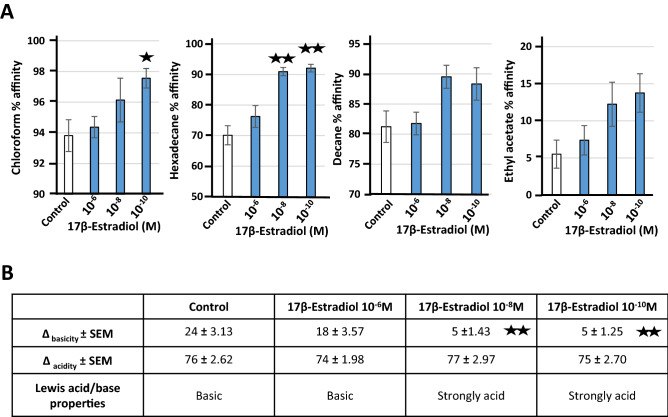
Effect of 17β-estradiol (10^–6^, 10^–8^, and 10^–10^ M) on the affinity for solvents and Lewis acid/base surface properties of *Lactobacillus crispatus* V4. (**A**) Partition between water and chloroform, water and hexadecane, water and decane, and water and ethyl acetate. (**B**) Lewis acid/base behavior of *L. crispatus* V4 with the two solvent couples hexadecane (HD)/chloroform (CH) and decane (DE)/ethyl acetate (EA). Values are the means ± SEM of three independent studies (^★^*p* < 0.05, ^★★^*p* < 0.01).

Given the key role of the *Lactobacilli* ultra-surface in the aggregation phenotype^16^, we studied the surface polarity of *L. crispatus* V4 by the microbial affinity to solvents (MATS) assay. This bacterium showed a high affinity for apolar solvents, particularly chloroform (94.0 ± 1.0%) and decane (81.0 ± 2.6%). Conversely, its affinity for ethyl acetate, a more polar solvent, was limited (6.0 ± 1.9%) (Fig. 4A). Exposure to 17β-estradiol induced a general increase in the affinity of *L. crispatus* V4 for solvents. This was particularly true for its affinity to hexadecane and chloroform, which showed a significant increase. As previously observed for the aggregation phenotype, the dose-related effect of 17β-estradiol was inverted in most cases and 10^–10^ 17β-estradiol had the greatest effect on the surface polarity of *L. crispatus* V4. Calculation of the Lewis acid/base ratio using the two solvent couples hexadecane/chloroform and decane/ethyl acetate showed the evolution of bacterial surface hydrophobicity to be associated with major changes in the electron donor/acceptor character. Indeed, although the surface of the control bacteria was basic, 17β-estradiol exposure made it highly acidic (*p* < 0.01) (Fig. 4B).

### 17β-estradiol has no effect on the membrane fluidity of *L. crispatus* V4

**Figure 5:**
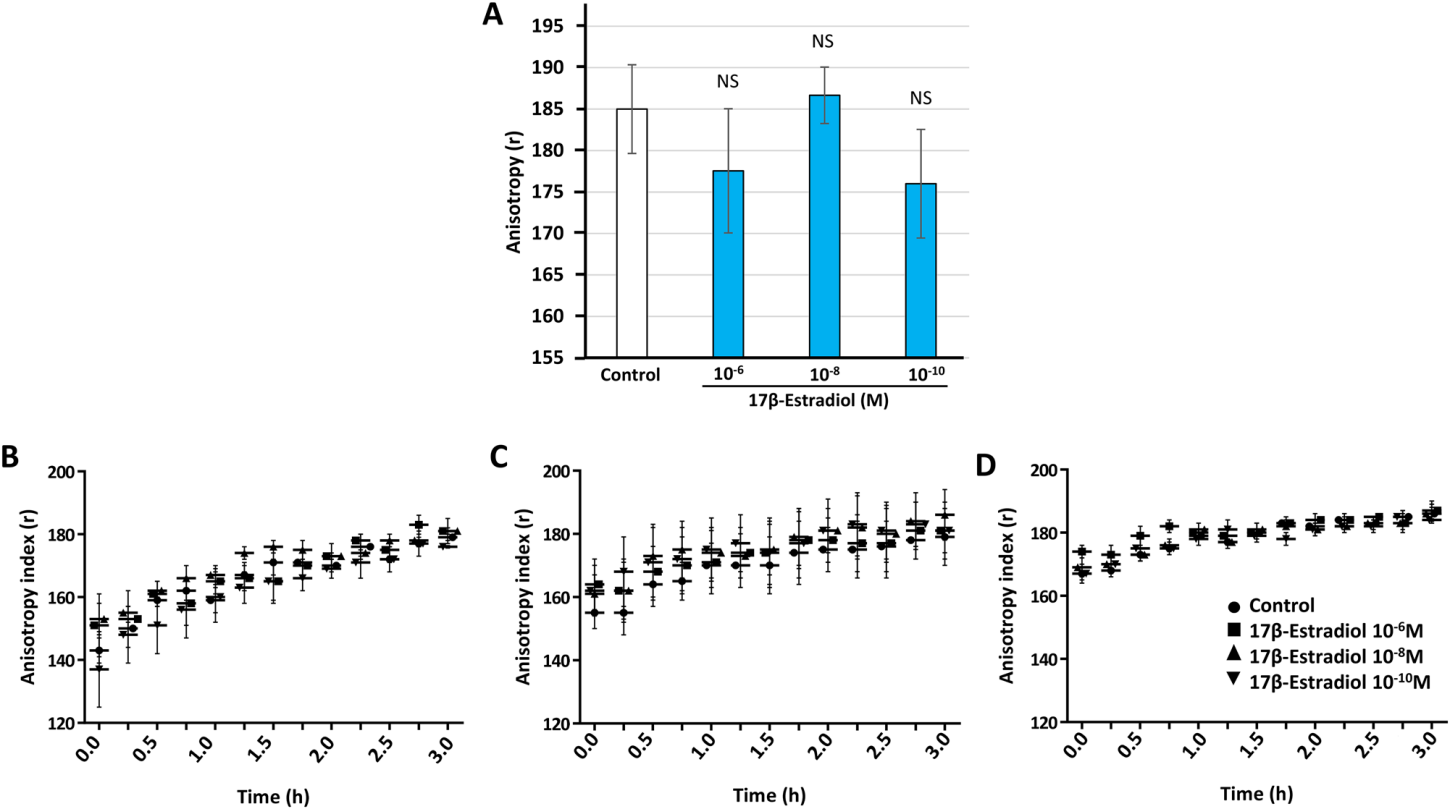
Effect of 17β-estradiol on *Lactobacillus crispatus* V4 membrane fluidity. (**A**) Membrane fluidity (anisotropy index) of *L. crispatus* V4 grown for 18 h in the absence (control) or presence of 17β-estradiol (10^–6^, 10^–8^, or 10^–10^ M). (**B**–**D**) Membrane fluidity (anisotropy index) of *L. crispatus* V4 exposed to 17β-estradiol (10^–6^, 10^–8^, or 10^–10^ M) after prior growth in MRS for (**B**) 6 h, (**C**) 12 h, or (**D**) 24 h. Values and curves are the means ± SEM of three independent studies (*NS* not significant).

We further investigated the potential effects of 17β-estradiol on the surface of *L. crispatus* V4 by studying the impact of the steroid on membrane integrity by fluorescence anisotropy. Membrane anisotropy can be measured after the incorporation of a fluorescent probe within the cell membrane and reflects its degree of organization. In a first set of experiments, we assessed this parameter in *L. crispatus* V4 exposed to 17β-estradiol (at 10^–6^, 10^–8^ or 10^–10^ M) for 18 h, from the onset of the experiment to the beginning of the stationary growth phase (Fig. 5A). We observed minor variations, but the overall organization of the bacterial membrane remained essentially unchanged. The bacterial membrane has been shown to evolve during the growth of planktonic cultures of *Staphylococcus aureus*, *Listeria monocytogenes*, and *Pseudomonas aeruginosa*, in particular, in terms of fatty-acids composition, which may lead to variations in membrane fluidity^17^. As 17β-estradiol is an amphiphilic molecule and can integrate into phospholipid membranes, it may affect membrane integrity^18^. Thus, we studied the effect of 17β-estradiol on the membrane fluidity of *L. crispatus* V4 after 6, 12, or 24 h growth before exposure to 17β-estradiol and the fluorescent probe DPH to label membranes^19–20^. The evolution of fluorescence anisotropy was subsequently monitored over 3 h. The membrane fluidity of bacteria collected during late exponential growth phase (6 h of growth) evolved during the 3 h of measurements from a mean “r” anisotropy index of 143 ± 4 to 179 ± 1 (Fig. 5B). However, 17β-estradiol had no effect on the changes in fluorescence anisotropy, and thus membrane integrity, at any concentration studied. Similar data were obtained for 12 or 24 h (early to late stationary growth phase) *L. crispatus* V4 (Fig. 5C,D). Thus, membrane stiffness increased over the 3 h of monitoring period but there was no effect of 17β-estradiol.

### 17β-estradiol alters the mean size distribution *of L. crispatus* V4 cells

**Figure 6:**
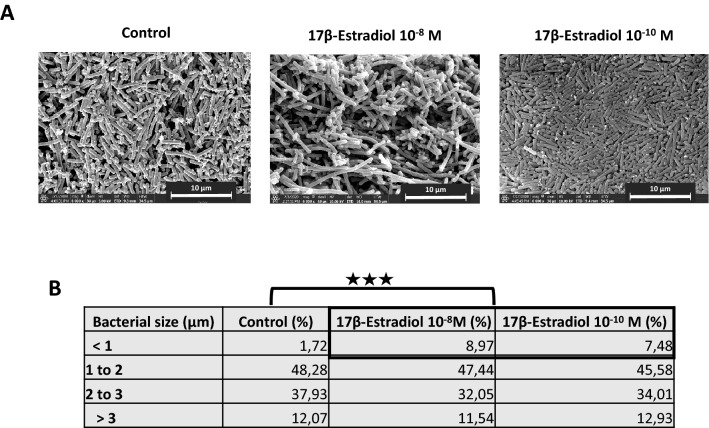
Effect of 17β-estradiol on the morphology of *Lactobacillus crispatus* V4. (**A**) Scanning electron microscope observations of *L. crispatus* V4 grown with or without 17β-estradiol (10^–8^ or 10^–10^ M). Scale bar = 10 µm. (**B**) Table showing the relative percentage of bacteria classified by size after culture with or without 17β-estradiol (10^–8^ or 10^–10^ M) (^★★★^*p* < 0.001).

Scanning electron microscopy (SEM) observations of *L. crispatus* V4 showed 17β-estradiol to have a limited impact on the general morphology of the bacteria (Fig. 6A). We then measured the mean size of the bacteria over 60 microorganisms under each condition and classified them from small (< 1 µm) to large (> 3 µm) to quantify the potential effect of 17β-estradiol. The percentage of medium to large size microorganisms remained unchanged, whereas the percentage of small size lactobacilli significantly increased after treatment with 10^–8^ and 10^–10^ M 17β-estradiol (+ 7.25 ± 0.06% and + 5.76 ± 0.043%, respectively, *p* < 0.001) (Fig. 6B).

### 17β-estradiol increases adhesion to vaginal mucosa epithelial cells and promotes biosurfactant production by *L. crispatus* V4

**Figure 7:**
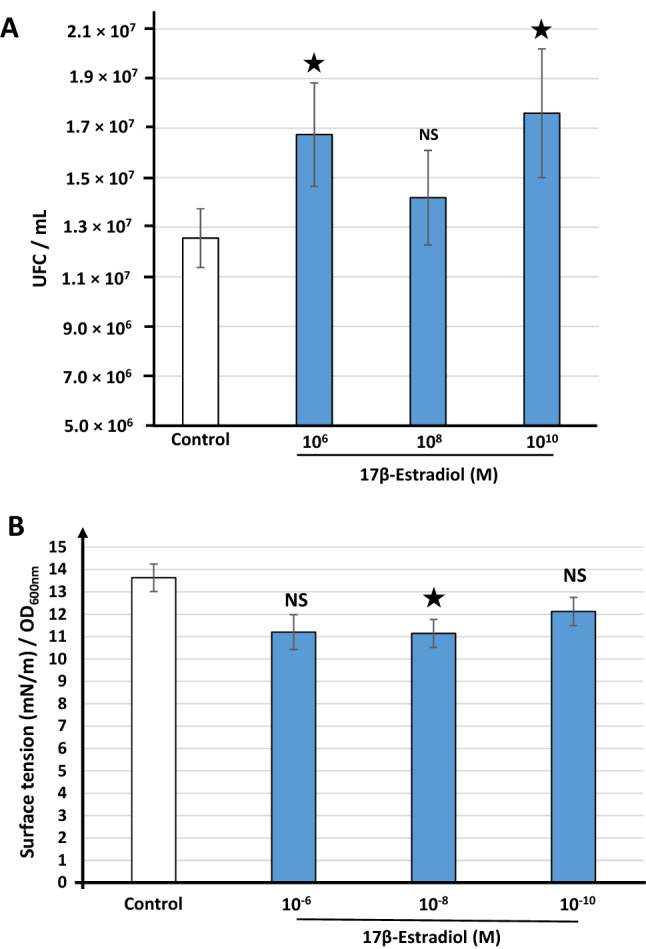
Effect of 17β-estradiol on *Lactobacillus crispatus* V4 adhesion to human VK2/E6E7 vaginal cells and biosurfactant production. (**A**) Effect of 17β-estradiol on the adhesion potential of *Lactobacillus crispatus* V4 grown in MRS medium to human VK2/E6E7 vaginal cells. (**B**) Surface tension of the water solution extracted from *L. crispatus* V4 cultures exposed or not to 17β-estradiol (10^–6^, 10^–8^, or 10^–10^ M) calculated from analysis of the curvature of hanging drops. The absence of an effect of 17β-estradiol on surface tension was controlled in preliminary studies. Values are the means ± SEM of three independent studies (*NS* not significant, ^★^*p* < 0.05).

We investigated the effect of 17β-estradiol on the adhesion of *L. crispatus* V4 to vaginal mucosa epithelial cells using the human vaginal VK2/E6E7 cell line. Exposure of the bacteria to 10^–6^ and 10^–10^ M 17β-estradiol significantly increased their adhesion to VK2/E6E7 cells (+ 33.22 ± 12.46 and + 40.09 ± 14.75%, respectively) (Fig. 7A). Bacteria exposed to 10^–8^ M 17β-estradiol showed the same tendency, but the difference was not statistically significant.

The adhesive properties of bacteria depend on the composition of their envelope, as well as that of secreted molecules, such as biosurfactants, which can adsorb to or insert into the bacterial membranes and modify their surface^21^. We investigated the effect of 17β-estradiol on biosurfactant production by growing *L. crispatus V4* colonies on MRS-agar with or without the steroid and then delicately scraping them off and extracting the obtained biomass in water. The solution, containing potential biosurfactants, was subsequently tested by the pendant drop method and the mean surface tension calculated from analysis of the curvature of the drops. We observed a tendency towards a decrease of the surface tension but 17β-estradiol showed no dose response (Fig. 7B). The effect of 17β-estradiol on the surface tension of the solution extracted from the *L. crispatus* V4 cultures was statistically significant at a concentration of 10^–8^ M.

### 17β-estradiol has a minor effect on *L. crispatus* V4 biofilm formation

**Figure 8:**
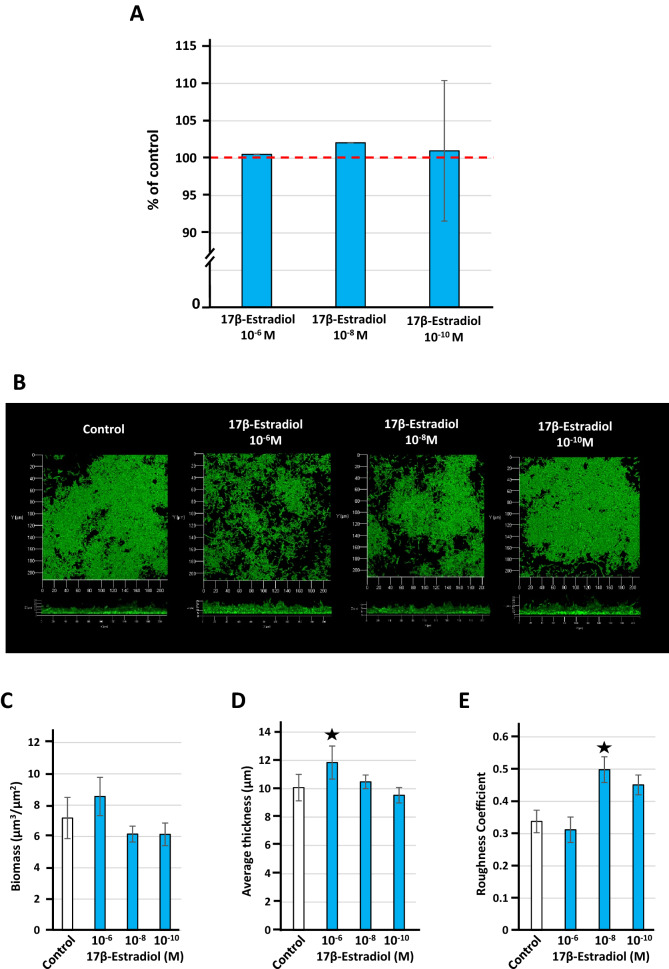
Effect of 17β-estradiol (10^–6^, 10^–8^, or 10^–10^ M) on biofilm formation of *Lactobacillus crispatus* V4. (**A**) Biofilm formation of bacteria grown in MRS medium, with or without 17β-estradiol, studied by the crystal violet staining assay. (**B**) Structure of the *Lactobacillus crispatus* CIP104459 biofilm formed in “simulating genital tract secretion” (SGTS) medium and visualized by confocal laser scanning microscopy. All figures are represented at the same scale and show top (X/Y) and lateral (X/Z mean thickness) views of the biofilms formed. Effect of 17β-estradiol on the calculated means biofilm biomass (**C**), thickness (**D**) and roughness (**E**). Results are representative of three independent experiments (^★^*p* < 0.05).

We studied biofilm formation by *L. crispatus* V4 by the crystal violet staining assay and confocal microscopy. No effect of 17β-estradiol on the biofilm formation activity of *L. crispatus* V4 was observed using the crystal violet staining assay (Fig. 8A). In order to investigate a potential effect of 17β-estradiol on the biofilm architecture, *L. crispatus* V4 biofilms were visualized by confocal laser scanning microscopy. Biofilm development in flat glass-bottom microtitration plates required growing the bacteria in a “simulating genital tract secretion” (SGTS) medium^22^, as previously described^8^. Under such conditions, *L. crispatus* V4 was capable to form a thin and heterogeneous biofilm (Fig. 8B). Treatment of the bacteria with 17β-estradiol had no visible effect on the biofilm structure. Biofilm images were then analyzed using COMSTAT2 software. This analysis revealed no significant variation in biomass (Fig. 8C). However, 10^–6^ M 17β-estradiol induced a minor (+ 17.54 ± 9.85%) but significant increase in mean biofilm thickness (*p* < 0.05) (Fig. 8D). No variations were observed at lower concentrations. The roughness coefficient of the biofilm also appeared to increase after exposure of the bacteria to 10^–8^ M 17β-estradiol, but the veracity of such an increase is still uncertain because of the heterogeneity of the biofilm (Fig. 8E).

## Discussion

Given the effect of 17β-estradiol on the adhesion of *L. crispatus* CIP104459 to vaginal mucosa cells^8^ and the need to develop new solutions to re-equilibrate the vaginal microbiota, it was necessary to verify whether this property is shared by different strains. Generally, the vaginal microbiota is composed of a single and almost clonal population of *Lactobacillus* and *L. crispatus* CIP104459 may be an exception. In addition, this strain was isolated in 1955 and is, thus, old, whereas bacteria, including *L. crispatus*, can rapidly evolve in response to host^23^ and environmental^24^ factors, especially by rearrangements within their genomic structure.

Here, we took advantage of the recent collection and draft genome sequencing of the vaginal strain *L. crispatus* V4, isolated in 2018 from a vaginal swab of a young healthy non-menopausal woman^11^. Comparison of the core genome of *L. crispatus* V4 to that of 122 *L. crispatus* strains show *L. crispatus* V4 and CIP104459 to be related to distinct and separated clusters and *L. crispatus* V4 appears to be a more typical vaginal strain. The core genomes of *L. crispatus* V4 and CIP104459 only show 97.42% average nucleotide identity and a 78.70% genome-to-genome distance. The two strains are therefore close to the limit of 96.8%, at which point they should be considered as different sub-species or even species^25^. In addition, alignment of the two whole genome sequences showed the core genome to be limited to 77.96% of the total genome and a high number the ORFs encode proteins specific to one or the other bacterial strains. There are large genomic variations in *L. crispatus* for genes encoding for glycosylation and glycogen degradation enzymes, which are essential for interaction with the vaginal mucosa^9^. Thus, we examined potential differences between our two strains. Principal genes involved in glycogen metabolism, including *glgX,* encoding for type 1pullulanase, are highly conserved between the two strains. Given the genomic distance between the two strains, we were surprised to observe that they expressed a highly similar number of proteins under our experimental conditions i.e., when bacteria were grown in MRS medium. However, the protein pools expressed by *L. crispatus* V4 and CIP104459 were notably different. Indeed, V4 appeared to express a high number of proteins also produced by CIP104459 (81.1%), as well as a series of strain-specific proteins. In addition, the expression of common proteins was generally higher in V4 than CIP104459, suggesting a higher metabolic activity for V4. However, none of the typical glycogen-degradation enzymes essential for interaction with the vaginal mucosa was detected in the proteome of either strain using our method. Medium composition, pH, and temperature strongly affect enzymes expression of *L. crispatus*^26^. MRS medium used in the present study and classically used for *Lactobacillus* culture contains yeast and meat extracts but probably not enough glycogen to induce expression of specific degradation enzymes.

Proteomic analysis also showed that 17β-estradiol has only a very minor effect on protein expression by *L. crispatus* V4. This result is coherent with the absence of an effect of 17β-estradiol on the growth kinetics of this strain, as previously observed with *L. crispatus* CIP104459^8^. Indeed, human hormones and neurohormones rarely affect the growth kinetics of human microbiota bacteria^27^. This is also coherent with clinical studies, which showed that the composition of the vaginal microbiota of women treated with estradiol-containing contraceptives is not significantly altered^28^.

All lactobacilli, and particularly *L. crispatus*, can spontaneously form aggregates^13^. In contrast to strain CIP104459, which shows only a limited potential for auto-aggregation^8^, *L. crispatus* V4 was capable to form dense aggregates, a phenotype that was increased by 17β-estradiol. The aggregation potential essentially depends on the outer surface properties of the bacteria, particularly S-layer composition^16^. Most lactobacilli have an S-layer, whereas the genes and protein sequences appear to be strain-specific^29^. This very likely explains the differences in strain behaviors in terms of biological activity, including aggregation and adhesion. Interestingly, flow cytometry studies showed the effect of 17β-estradiol to correlate with increased bacterial surface heterogeneity, i.e., presumably with S-layer re-arrangement. These observations are coherent with the results of the MATS studies, which showed that 17β-estradiol induced significant changes in the surface polarity of *L. crispatus* V4. The affinity of *L. crispatus* V4 for all solvents, particularly decane and hexadecane, was markedly increased by exposure to 17β-estradiol, showing globally higher surface hydrophobicity of the bacterium. In parallel, the Lewis acid/base properties of *L. crispatus* V4 evolved from basic to strongly acidic. S-layer proteins are generally hydrophobic^30^ and this result suggests reinforcement of the bacterial crystalline envelope. Such behavior of *L. crispatus* V4 is entirely different from that observed with the strain CIP104459, in which 17β-estradiol has no, or a very limited, impact on aggregation and surface polarity^8^. However, it should be noted that, in sharp contrast to V4, CIP104459 shows low affinity to solvents and its surface is much more polar, suggesting that the composition of its S-layer is different or that it is incomplete or absent. The impact of 17β-estradiol on bacterial membrane fluidity was also very different. *L. crispatus* V4 showed only minor changes after exposure to 17β-estradiol, whereas *L. crispatus* CIP104459 reacts to the steroid by a significant decrease in the anisotropy index, indicating membrane alterations^8^. As an amphiphilic molecule, 17β-estradiol can integrate into membranes and destabilize their structure^18^. This phenomenon has been particularly observed in Gram-negative bacteria, such as *Helicobacter pylori*^18^ and *Pseudomonas aeruginosa*^31^. In this regard, the absence of an effect of 17β-estradiol on the membrane integrity of *L. crispatus* V4 can be explained by the higher protection conferred by a complete outer S-layer envelope. Nevertheless, the lack of an effect on membrane fluidity of 17β-estradiol, administered during the entire growth phase or at various periods during growth, reinforces the hypothesis that, as previously suggested^8^, 17β-estradiol likely acts on the physiology of *L. crispatus* through a signal transduction-dependent mechanism and not by non-specific membrane integration, at least at the concentrations studied. *L. crispatus* V4 exposed to 17β-estradiol showed, as noted for CIP104459, a decrease in the mean cell size. We also observed an increase of *L. crispatus* V4 adhesion to VK2/E6E7 human vaginal cells. In addition to aggregation, the S-layer plays a central role in bacterial morphology and adhesion to various targets^32–33^. However, S-layer composition was not the unique parameter affected by 17β-estradiol. As revealed by measurement of the surface tension of aqueous extracts of *L. crispatus* V4 colonies grown in the presence of 17β-estradiol, surface tension slightly decreased, suggesting an increase in biosurfactant production. Unexpectedly, the effect of 17β-estradiol on cell adhesion and biosurfactant activity was not no dose-related and there were no significant differences between values obtained after exposure to the hormone. This was also true for *L. crispatus* V4 aggregation, which showed an inverted dose-relationship for 17β-estradiol. One potential explanation is that the surfactant activity of biosurfactants is not directly related to their concentration. Indeed, their properties and interactions with the environment are different when under or above the critical micellar concentration^34^. Added to the fact that 17β-estradiol must be at the appropriate concentration to favor biosurfactant release and aggregation or adhesion to the target cells, as suggested for *L. crispatus* CIP104459^8^, the relationship between bacterial phenotype and dose may not be linear. As previously observed, the impact of 17β-estradiol on *L. crispatus* V4 biofilm formation was marginal. However, the vaginal epithelium is covered by cervicovaginal mucus^35^ and *Lactobacillus* has no need to synthesize extracellular matrix to implant. Pathogens involved in bacterial vaginosis develop in biofilms^36^. Conversely, the installation and persistence of *L. crispatus* in mucosa appears to depend essentially on its aggregation phenotype^37^ and, as demonstrated herein, 17β-estradiol stimulates this process.

These results are of importance, as they show that, although the *L. crispatus* V4 and CIP104459 strains belong to different genetic clusters and have different surface properties, the effect of 17β-estradiol on adhesion to vaginal mucosa cells is preserved in both. Thus, 17β-estradiol may have a similar effect on most of *L. crispatus* strains by promoting adhesion to vaginal cells or adsorption in the vaginal mucosa, without modifying the population dynamics. These observations are coherent with clinical studies showing that the vaginal microflora tends to re-equilibrate in favor of *L. crispatus* and other normal flora *Lactobacillus* for women under treatment with 17β-estradiol, such as administration during in vitro fertilization protocols^38^. Give the risk inherent to the carcinogenic activity of estradiol^39^, 17β–estradiol is not the ideal candidate drug for the development of a product to re-equilibrate the vaginal microbiota, but there are many other phytoestrogens that may show more specific activity on *Lactobacillus*.

## Methods

### Bacterial strain and culture conditions

Vaginal *Lactobacillus crispatus* V4 was obtained from a swab of a non-menopausal woman^11^. The sample was collected by the CRO Bio-EC (Longjumeau, France) and in agreement with the French and EU ethical guidelines (ARS Biomedical Research Agreement N°2012-12-010, Bioethics Agreement DC-2008-542). This strain was characterized by total proteome identification using a Bruker Autoflex III MALDI-TOF/TOF mass spectrometer coupled to the algorithmic software Biotyper. The draft genome sequence of *L. crispatus* V4 was determined using an Illumina MiSeq platform and deposited in the DDBJ/ENA/GenBank under accession number SRLG00000000^11^. The bacterium was grown anaerobically in De Man, Rogosa and Sharpe (MRS) medium (VWR, Fontenay-sous-Bois, France) at 37 °C under static conditions. Pre-cultures were prepared anaerobically in the same medium for 48 h to reach the stationary growth phase. Bacterial density of the suspension was determined by measuring the OD_600nm_ using a ThermoSpectronics spectrophotometer (Cambridge, UK). The absence of contamination was controlled by plating on Petri dishes of MRS-Agar (VWR, Fontenay-sous-Bois, France).

For growth kinetic studies, bacteria were grown in microplates under anoxic conditions for 48 h at 37 °C with shaking (360 rpm) for 10 s before each measurement using a Tecan multimode microplate reader (Tecan Group Ltd., Männedorf, Switzerland). Growth curves were generated by the automatic measure of the OD_600 nm_ every 30 min.

### Tested molecule

17β-estradiol (Sigma-Aldrich, Saint-Quentin-Fallavier, France) is poorly soluble in water. Thus, a stock solution was prepared in 100% ethanol and used to prepare final dilutions in MRS. The final concentration of ethanol in MRS was 0.1% v/v for all dilutions, as well as the control. The absence of an effect of ethanol 0.1% on the growth kinetics of *L. crispatus* V4 was verified in preliminary studies.

### Genomic analysis

Genome sequences of *L. crispatus* V4 and 122 other *L. crispatus* strains (including CIP104459), in addition to *Leuconostoc mesenteroides* subsp. *mesenteroides* ATCC8293 (used as an outlier to root the phylogenetic tree), were retrieved from RefSeq on November 3, 2020. The average nucleotide identity (ANI) and in silico DNA-DNA hybridization (*is*DDH; using GGDC formula 2 and *is*DDH estimates, based on identities/high-scoring segment pairs lenght) approaches were used to determine the genetic relationship between the 124 genome sequences using Pyani v.0.2.7^40^ and GGDC v. 2.1 (Genome-to-Genome Distance Calculator; http://ggdc.dsmz.de/distcalc2.php). MAUVE v.2.4.0^41^ was used to align V4 and CIP104459. Pangenome analysis was conducted on the V4 and CIP104459 genome sequences using Roary v.3.13^42^.

### Proteomic analysis

Bacteria were collected by centrifugation (7500×*g*, 10 min) in early stationary phase (18 h culture), rinsed three times with Tris–HCl buffer (20 mM) pH 7.4 and resuspended in the same buffer. Prior to the lysis step, a protease inhibitor solution was added (1-× final concentration, complete EDTA-free Protease Inhibitor Cocktail, Roche, Sigma-Aldrich, Saint-Quentin-Fallavier, France). For lysis, the same volume of bacterial suspension (1 mL) was distributed into 2 mL tubes containing 0.1-mm diameter glass beads (VK01-2 mL, P000914-LYSK0-A, Precellys, Bertin Technologies Montigny-le-Bretonneux, France). The cells were mechanically lysed three times by agitation at 6800 rpm for 30 s, with a break of 30 s between each cycle, using a homogenizer (Precellys 24, Bertin Technologies Montigny-le-Bretonneux, France). The temperature was maintained at 4 °C during cell lysis using an integrated dry ice cooling module (Cryolys Evolution). The samples were then treated with benzonase (Benzonase Nuclease E1014-5KU, Sigma Aldrich, Darmstadt, Germany) and 2 mM of MgCl_2_ (REF 036226, Alfa Aesar) for 30 min at 4 °C. The beads were removed by a first centrifugation (13,000×*g*, 2 min, 4 °C) and then the cellular debris by a second (13,000×*g*, 18 min, 4 °C). The protein concentration in the supernatant was estimated by the Bradford's method. The samples (50 µL) were mixed with 1.2 mL of Bradford's solution (Pierce Coomassie Plus Protein Assay Reagent 23238 Thermo Fisher scientific, Waltham, Massachusetts) and incubated for 15 min in the dark at room temperature before measurement of the OD_595nm_. A standard curve was made using bovine serum albumin.

Sample preparation and protein digestion were performed as previously described^43^. For each extract, 25 μg of proteins was loaded onto a 7% polyacrylamide gel (Acrylamide/Bis-Acrylamide 30% [29:1], Sigma-Aldrich) and migration performed for a short period (90 min at 10–20 mA/gel). After Coomassie blue staining, the revealed protein bands were excised and first immersed in reductive buffer (5 mM DTT, Sigma-Aldrich) and then an alkylated buffer (20 mM iodoacetamide, Sigma-Aldrich). After washing, the gel bands were digested with 1 μg of trypsin (Promega, Madison, WI) overnight at 37 °C. Then, several steps of peptide extraction were performed using acetonitrile (Fisher, Hampton, NH) and the peptides dried and stored at − 20 °C.

All experiments were performed using an LTQ-Orbitrap Elite mass spectrometer coupled to an Easy nLC II system (both from Thermo Scientific). Samples were injected onto an enrichment column (Acclaim PepMap100, Thermo Scientific). The separation was achieved with an analytical column needle (NTCC-360/100-5-153, NikkyoTechnos). The mobile phase consisted of H_2_O/0.1% fluorhydric acid (FA) (buffer A) and acetonitrile(CAN)/ 0.1% FA (buffer B). Tryptic peptides were eluted at a flow rate of 300 nL/min using a three-step linear gradient: from 2 to 40% B over 76 min, from 40 to 80% B over 4 min, and 100% B for 10 min. The mass spectrometer was operated in positive ionization mode with the capillary voltage set to 1.7 kV and the source temperature 275 °C. The samples were analyzed using CID (collision-induced dissociation) method. The first scan (MS spectra) was recorded in the Orbitrap analyzer (R = 60,000) with a mass range of m/z 400–1800. Then, the 20 most intense ions were selected for MS2 experiments. Singly charged species were excluded from the MS2 experiments. Dynamic exclusion of already fragmented precursor ions was applied for 30 s, with a repeat count of 2, a repeat duration of 30 s, and an exclusion mass width of ± 5 ppm. The precursor isolation width was 2 m/z. Fragmentation occurred in the linear ion trap analyzer with a normalized collision energy of 35. All measurements in the Orbitrap analyzer were performed with on-the-fly internal recalibration (lock mass) at an m/z of 445.12002 (polydimethylcyclosiloxane).

Raw data files were processed using Proteome Discoverer 1.4 software (Thermo Scientific). Peak lists were searched using MASCOT search software (Matrix Science) against the Uniprot database. Database searches were performed using the following parameters: two missed trypsin cleavage sites allowed, variable modifications: carbamidomethylation on cysteine and oxidation on methionine. The parent-ion and daughter-ion tolerances were 5 ppm and 0.35 Da, respectively. The false discovery rate (FDR) threshold for identifications was set to 1% (for proteins and peptides).

For protein quantification, a label-free experiment was performed as previously described by Kentache et al*.*^44^. Briefly, after MS analysis, raw data were imported into Progenesis LC–MS software (Nonlinear Dynamics, version 4.0.4441.29989, Newcastle, UK). For comparison, one sample was set as a reference and the retention times of all other samples within the experiment were aligned. After alignment and normalization, statistical analysis was performed for one-way ANOVA calculations. For quantitation, peptide features with p- and q-values < 0.05 and a power > 0.8, were retained. MS/MS spectra from selected peptides were exported for peptide identification by Mascot (Matrix Science, version 2.2.04). Database searches were performed using the following parameters: one missed trypsin cleavage site allowed, variable modifications: carbamidomethylation of cysteine and oxidation of methionine. Mass tolerances for precursor and fragment ions were set at 5 ppm and 0.35 Da, respectively. FDRs were calculated using a decoy-fusion approach in Mascot (version 2.2.04). Identified peptide-spectrum matches with a − 10logP value of ≥ 13 were kept. Mascot search results were imported into Progenesis. For each growth condition, the total cumulative abundance of the protein was calculated by summing the abundances of peptides. Proteins identified with < 2 peptides were discarded. Analyses were performed in triplicate on independently prepared samples.

### Bacterial aggregation studies

*L. crispatus* V4 grown in microplates showed the formation of dense aggregates. The aggregation potential of *L. crispatus* V4 was quantified by sedimentation^14^. Bacteria were grown in MRS at 37 °C for 18 h under anoxic static conditions with or without 17β-estradiol and harvested by centrifugation (7500×*g*, 10 min, 20 °C). After washing twice in phosphate buffered saline (PBS, Lonza, Thermo Fisher Scientific, Waltham, Massachusetts, USA) and resuspension in 10 mL of the same medium, the bacterial suspension was vortexed for 1.5 min. The OD_600nm_ of the suspension was measured 30 min later using a Thermo Fisher Scientific spectrophotometer (Waltham, Massachusetts, USA). The percentage of auto‐aggregation was calculated as:

$$ \% \, {\text{auto-aggregation}} =  \left( {\left[ {\left( {{\text{OD}}_{0{{\rm min}} } \, - {{\text{ OD}}}_{30{{\rm min}} } } \right)/{{\text{OD}}}_{0{{\rm min}} } } \right]} \right)/{{\text{OD}}}_{0{{\rm min}} } \times 100 $$

where OD_0min_ is the initial OD_600nm_ at T = 0 and OD_30min_ is the final OD_600nm_ after 30 min.

The aggregation potential and morphology of *L. crispatus* V4 were further studied by flow cytometry using a CytoFlex S flow cytometer (Beckman Coulter Life science, Indianapolis, USA) and CytExpert software. Control and 17β-estradiol treated 18-h bacterial cultures were harvested by centrifugation (7500×*g*, 10 min, 20 °C) and resuspended in PBS. Bacteria were immediately aliquoted, distributed in 96 wells microplates (Thermo Fisher Scientific, Waltham, Massachusetts, USA) and maintained under static conditions for 30 min before flow cytometry analysis. A minimum of 10,000 events at OD_488 ± 4 nm_ (SSC channel) and OD_525 ± 20 nm_ (FSC channel) was recorded at a flow rate of 10 µL min^−1^ in each condition. Data were analyzed using Cytexpert software. The aggregates correspond to the fraction of events appearing in the Q1-UR quarter of the graph (red zone). Particles size is given by the FSC-A (horizontal) axis. Isolated bacteria appear in the Q1-UL and Q1-LL zones. Granulometry (surface heterogeneity) is plotted in the SSC-A (vertical) axis. Increased granulometry appears in the Q1-UL and Q1-UR areas of the graph. Measures were realized in triplicate.

### Investigation of bacterial ultra-surface properties

*L. crispatus* V4 ultra-surface properties were investigated by determining the surface polarity and Lewis acid–base characteristics using the microbial adhesion to solvents (MATS) technique^45^. Bacteria grown for 18 h, with or without 17β-estradiol, were collected as already described. Traces of culture medium were removed by rinsing twice in PBS. Four solvents were used chloroform, hexadecane, decane, and ethyl acetate. For each test, 1.2 mL of bacterial suspension at OD_400nm_ = 0.8 was mixed with 0.2 mL solvent. After incubation for 15 min and separation of the two phases, the OD_400nm_ of the aqueous phase was measured. Bacteria divide into the two compartments based on their surface affinity for water or organic solvents. The affinity was calculated using the equation:

$$ \% \, {{\text{solvent }}}\,{{\text{affinity }}} = (1 - {{\text{A}}})/{{\text{A}}}0  \times 100 $$

where [AO] is the OD_400nm_ of the aqueous phase without solvent and [A] the OD_400nm_ of the aqueous phase after exposure to the solvent. All experiments were conducted at least in triplicate.

### Evaluation of the degree of bacterial membrane organization

Bacterial membrane fluidity, and therefore the degree of organization, was investigated by measurement of the fluorescence anisotropy, as previously described^19^. *L. crispatus* V4 was grown with or without 17β-estradiol for 18 h and harvested by centrifugation (7500×*g*, 10 min), washed twice in 10 mM MgSO_4_ and stained with the fluorescent probe 1,6-diphenyl-1,3,5-hexatriene (DPH) (Sigma-Aldrich, Saint-Quentin-Fallavier, France) at 4 mM in tetrahydrofuran using a probe to sample ratio of 1/1000. The probe was allowed to incorporate into the membranes by incubating for 30 min in the dark at 37 °C. Then fluorescence polarization was continuously measured using a temperature-controlled Spark 20 M multimode microplate reader. The excitation and emission wavelengths of the probe were 365 and 425 nm, respectively. Each measurement was performed in triplicate. The membrane anisotropy index (r value) was calculated according to Lakowicz^46^. Data were analyzed using SparkControl software 2.1 (Tecan Group Ltd., Männedorf, Switzerland). Increases in anisotropy values indicate a decrease in membrane fluidity and vice versa. In a second series of experiments, bacteria grown for 6, 18 or 24 h in the absence of treatment were collected and subsequently exposed to 17β-estradiol or control medium in parallel with incorporation of the fluorescent probe. The anisotropy index was then continuously monitored over 3 h.

### Scanning electron microscopy

The morphology of *L. crispatus* V4 was studied by scanning electron microscopy (SEM) using a TENEO VolumeScope microscope (FEI, Hillsboro, OR, USA). Bacteria grown for 18 h with or without 17β-estradiol were harvested by centrifugation (7500×*g*, 10 min) and fixed by immersion in 1 mL of 2.5% glutaraldehyde in 1 M PBS, pH 7.1, for 1 h. Samples were prepared by hexamethyldisilazane treatment and coating in a LEICA EM ACE600 sputter coater (Wetzlar, Germany) with a 25 nm thick layer of platinum alloy, as previously described^47^. The SEM was operated at 10 kV.

### Bacterial adhesion to vaginal cells

Adhesion of *L. crispatus* V4 was studied using the VK2/E6E7 mucosa vaginal cells line (ATCC CRL-2616). These cells were originally collected from the vaginal mucosal of a healthy pre-menopausal women. They were grown and propagated using keratinocyte serum-free medium (KSFM) (Thermo Fisher Scientific, Waltham, Massachusetts, USA), supplemented with 0.05 mg/mL bovine pituitary extract, 0.1 ng/mL human recombinant EGF, and 44.1 mg/mL calcium chloride, as recommended by the provider.

VK2/E6E7 cells (80% confluence) were exposed to *L. crispatus* V4 at a multiplicity of index (MOI) of 100 bacteria/cell. Prior to infection, bacteria grown with or without 17β-estradiol for 18 h were collected by centrifugation, rinsed twice with PBS to remove any trace of the steroid, and resuspended in KSFM without antibiotics. After 1 h of interaction with the bacteria, the medium was removed and the VK2/E6E7 cells rinsed carefully with KSFM without antibiotics to withdraw all planktonic bacteria. Then, the vaginal cells were lysed by the addition of 0.1% triton X-100 in PBS. Preliminary studies showed that the cultivability of *L. crispatus* V4 was not affected by such treatment. The bacterial solution was then diluted in MRS medium and plated on MRS agar Petri dishes. The number of adherent bacteria was determined by the direct counting of *L. crispatus* colonies grown after 48 h at 37 °C under anoxic conditions.

### Investigation of biosurfactant production

*L. crispatus* V4 biosurfactant production was studied using the pendant drop method. As described by Meylheuc et al.^48^, the bacterial mat of *L. crispatus* V4 grown on normal or 17β-estradiol supplemented MRS-Agar Petri dishes was scraped off and resuspended in 15 mL Volvic water (selected for its neutral surface tension value). The solution was homogenized by vortexing for 3 min and centrifuged twice for 30 min (10,000×*g,* 4 °C) to remove all debris. The biosurfactant containing supernatant was collected and stored at 4 °C. Measurements were performed using a DSA30 tensiometer temperature controlled drop shape analyser (Kruss, Hamburg, Germany). The surface tension was calculated from the drop shape curvature analysis based on the Young–Laplace equation using the tensiometer drop shape analysis software^49^. Surface tension values were merged using the OD_600nm_ of the bacterial suspension collected by scrapping of the Petri dishes to account for the variations due to the biomass collected.

### Biofilm formation study

The impact of 17β-estradiol on biofilm formation of *L. crispatus* V4 was evaluated by the crystal violet staining assay and confocal laser scanning microscopy. Crystal violet studies were performed following a procedure adapted from O’Toole^50^. After pre-culture for 48 h in MRS medium, the bacterial solution was adjusted to an OD_600nm_ = 0.1 and supplemented with 17β-estradiol to reach a final concentration of 10^–6^, 10^–8^, or 10^–10^ M. The same volume (1 mL) was distributed into each well of flat glass bottom 24-well polystyrene plates (Falcon^®^, Durham, USA). Plates were incubated at 37 °C without agitation for 48 h in a Whitley A85 anaerobic Workstation. After incubation, non-adhered bacteria were removed by aspiration of the medium and rinsing with physiological water (0.9% NaCl). The crystal violet solution (0.1% w/v in sterile pure 18.2 MΩ water) was distributed in microwells and allowed to stain the biofilm for 10 min. Then, the excess of dye was removed by washing with pure 18.2 MΩ water. Absolute ethanol was added to dissolve the crystal violet adsorbed in the biofilm matrix and onto bacteria and the OD_595nm_ of the solution measured using an automated Te-Cool plate reader (Tecan Group Ltd., Männedorf, Switzerland).

*L. crispatus* V4 is unable to form biofilms on glass bottom microplates in MRS medium. To visualize the biofilm structure by confocal microscopy, *L. crispatus* V4 was grown in a special medium for vaginal microflora lactobacilli, designated as “simulating genital tract secretion” (SGTS) medium^20^. 17β-estradiol, or an equivalent volume of ethanol in water, was added from the onset of the culture to the end of the experiment. The absence of effect of 17β-estradiol on *L. crispatus* V4 growth in SGTS medium was verified in preliminary studies (Suppl. Figure 1B). For confocal microscopy, pre-cultures were performed in normal MRS medium. At the end, bacteria were collected by centrifugation (7500×*g*, 10 min), re-suspended in normal (control) or 17β-estradiol supplemented SGTS medium at an OD_600nm_ = 0.1 and distributed (1 mL aliquots) into 24-well glass flat bottom microplates (Sensoplate, Greiner Bio-One, Germany). Biofilm was allowed to develop for 48 h under static conditions in an anoxic chamber (DW scientific, Bingley, UK). Nonadherent bacteria were removed by rinsing twice with PW and the biofilms stained with SYTO 9 Green Fluorescent Nucleic Acid Stain (Thermofisher, Waltham, Massachusetts, USA). Biofilms were visualized under an LSM 710 inverted confocal laser scanning microscope (Zeiss, Marly-le-Roi, France) equipped the Zen 2009 software package (version 12.0.1.362). The average biofilm thickness (μm), mean biomass volume (μm^3^/μm^2^), and roughness coefficient were calculated over a minimum of 30 observations for each condition using COMSTAT2 software. All experiments were repeated at least of three times.

### Statistical analysis

The statistical significance of experimental values was evaluated using the Prism GraphPad online tool (https://www.graphpad.com/quickcalcs/ttest1/). Data were analyzed using unpaired (two sample) two-tailed *t* tests to calculate *p* values.

### Ethics approval and consent to participate

Not applicable, this study did not involve a clinical trial.

### Consent for publication

All authors have read and approved the manuscript. LMSM accepts to be responsibility for the publication fees.

## Supplementary Information

Supplementary Table 1.

Supplementary Table 2.

Supplementary Table 3.

Supplementary Information.

Supplementary Figure 1.

## Data Availability

All raw experimental data are available upon simple request to the authors.
