# Supplementary material for: Variability of the response of human vaginal Lactobacillus crispatus to 17β-estradiol
Source: Sci Rep. 2021 Jun 1;11:11533. doi: 10.1038/s41598-021-91017-5 (PMC8169910; doi:10.1038/s41598-021-91017-5)
Supplement: Supplementary file 4 — Supplementary Information. [file 41598_2021_91017_MOESM4_ESM.docx]

***Supplementary data title page:***

**Variability of the response of human vaginal *Lactobacillus crispatus* to 17β-estradiol**

Maximilien CLABAUT, Amine M. BOUKERB, Amine Ben MLOUKA, Amandine SUET, Ali TAHRIOUI, Julien VERDON, Magalie BARREAU, Olivier MAILLOT, Agathe LE TIRANT, Madina KARSYBAYEVA, Coralie KREMSER, Gérard REDZINIAK, Cécile DUCLAIROIR-POC, Chantal PICHON, Julie HARDOUIN, Pascal COSETTE, Sylvie CHEVALIER, Marc G.J. FEUILLOLEY

**Supplementary Table 1**. Comparison of genome identity values of *Lactobacillus crispatus* V4 genome (green box) with 122 vaginal *Lactobacillus* strains for which the genome was accessible in the NCBI RefSeq database, including the reference strain *Lactobacillus crispatus* CIP104459 (blue box). The genome of an unrelated bacterial species, presently *Leuconostoc mesenteroides* subspecies Mesenteroides ATCC8293 was used as a reference.

**Supplementary Table 2**. Complete list of proteins identified in the total *Lactobacillus crispatus* V4 proteome classified by functions.

**Supplementary Table 3.** List of proteins expressed by *Lactobacillus crispatus* V4 and *Lactobacillus crispatus* CIP104459 with a > 2 fold change classified by function. Up-regulated proteins in *L. crispatus* V4 compared to those in CIP104459 are indicated by a green box. Downregulated proteins are indicated by a red box.

**Supplementary Figure 1.** Growth kinetics of *Lactobacillus crispatus* V4, with or without 17β-estradiol (10^-6^, 10^-8^, or 10^-10^ M). (A) Bacteria grown under anaerobic conditions at 37°C for 24h in MRS broth. (B). Bacteria grown under anaerobic conditions at 37°C for 24h in SGTS medium. All data are presented as the mean ± SEM of four independent experiments.
