# Supplementary material for: Variability of the response of human vaginal Lactobacillus crispatus to 17β-estradiol
Source: Sci Rep. 2021 Jun 1;11:11533. doi: 10.1038/s41598-021-91017-5 (PMC8169910; doi:10.1038/s41598-021-91017-5)
Supplement: Supplementary file 5 — Supplementary Figure 1. [file 41598_2021_91017_MOESM5_ESM.pptx]

## Slide 1
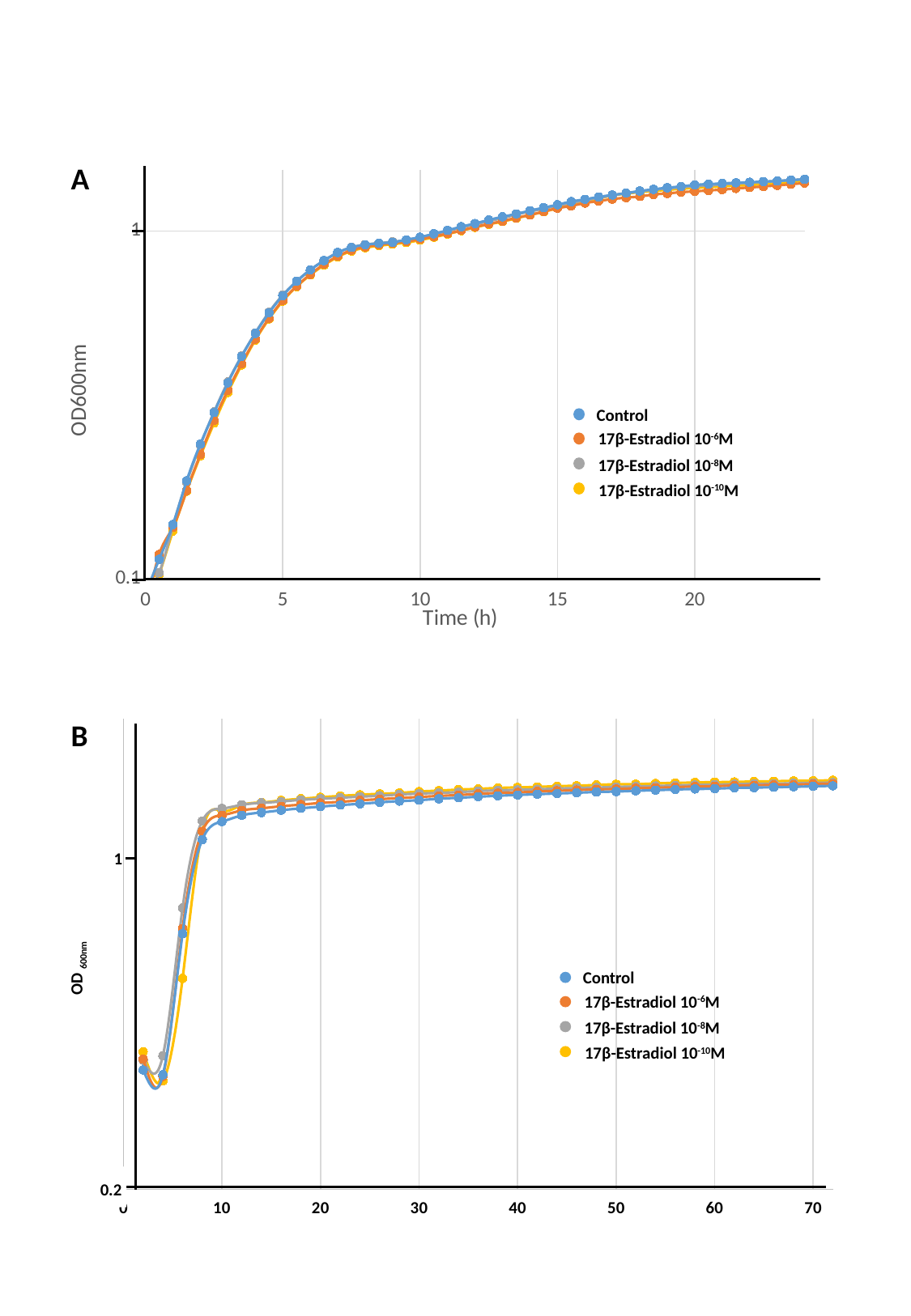

### Chart
| Category | Control V4 | V4 + Estradiol 10-6M | V4 + Estradiol 10-8M | V4 + Estradiol 10-10M |
|---|---|---|---|---|A
Control
17β-Estradiol 10-6M
17β-Estradiol 10-8M
17β-Estradiol 10-10M
### Chart
| Category | Control | E 10-6 M | E 10-8M | E 10-10M |
|---|---|---|---|---|B
1
OD 600nm
Control
17β-Estradiol 10-6M
17β-Estradiol 10-8M
17β-Estradiol 10-10M
0.2
